# Interferon*β*-1b Induces the Expression of RGS1 a Negative Regulator of G-Protein Signaling

**DOI:** 10.1155/2010/529376

**Published:** 2011-01-17

**Authors:** Tiffany Tran, Pedro Paz, Sharlene Velichko, Jill Cifrese, Praveen Belur, Ken D. Yamaguchi, Karin Ku, Parham Mirshahpanah, Anthony T. Reder, Ed Croze

**Affiliations:** ^1^Bayer HealthCare, Applied Research, Richmond, CA 94804, USA; ^2^Department of Neurology, University of Chicago, Chicago, IL 60687, USA; ^3^Translational Research, Specialty Medicine, Global Medical Affairs, Bayer HealthCare, US Innovation Center, 455 Mission Bay Boulevard South, San Francisco, CA 94158, USA

## Abstract

We present evidence of a link between interferon*β*-1b (IFN-*β*) and G-protein signaling by demonstrating that IFN-*β* can induce the expression of the negative regulator of G-protein signaling 1 (RGS1). RGS1 reduces G-protein activation and immune cell migration by interacting with heterotrimeric G-proteins and enhancing their intrinsic GTPase activity. In this study, IFN-*β* treatment resulted in the induction of RGS1 in peripheral blood mononuclear cells (PBMCs), monocytes, T cells, and B cells. Induction of RGS1 by IFN-*β* was concentration dependent and observed at both the RNA and protein level. Other members of the RGS family were not induced by IFN-*β*, and induction of RGS1 required the activation of the IFN receptor. In addition, RGS1 induction was observed in PBMCs obtained from IFN-*β*-treated multiple sclerosis patients suggesting a possible, as yet unexplored, involvement of G-protein regulation in disease treatment. The upregulation of RGS1 by IFN-*β* has not been previously reported.

## 1. Introduction

A cornerstone of the inflammatory response is the highly coordinated interplay of inflammatory regulators and immune cell migration to sites of injury or infection [1–3]. Immune cells act in concert to mitigate adverse responses resulting from exposure to toxins, bacteria, viruses, or various pathogens [4]. Regulation of innate and adaptive immune responses is a tightly controlled process involving a plethora of different cell types that interact with and respond to changing levels of immune mediators. Migration and accumulation of immune cells at sites of injury or infection eventually result in the resolution of inflammation and repair of damaged tissue. However, dysregulation of immune surveillance and activation of immune responses toward self-antigens can lead to adverse effects including autoimmune diseases such as rheumatoid arthritis [5], inflammatory bowel disease [6], and relapsing-remitting multiple sclerosis (MS) [7].

Cytokines and chemokines are members of two different families of immune mediators that each play an indispensable role in the control and regulation of an immune response. They differ based on their chromosome location, cell specificity, protein structure and function, cell surface receptor, and cellular signaling pathways. The immune system makes use of these immune mediators to coordinate and efficiently direct immune responses [8, 9]. 

Interferons (IFNs) are part of the cytokine family of proteins and are comprised of two different groups: type I (IFN-*α*, -*β*) and type II (IFN-*γ*) [9]. Type I and type II IFNs differ in structure, cell surface receptor, and, to some extent, cellular signaling. Type II IFNs have been associated with proinflammatory effects in contrast to type I IFNs which appear anti-inflammatory [9]. IFN-*α* and IFN-*β* are located on different chromosomes and exhibit a high degree of homology at both the amino acid and nucleotide levels. Type I IFNs bind to the same heterodimeric receptor although receptor assembly and signaling events can differ [10, 11]. Activation of the IFN receptor leads to a cascade of intracellular signaling events involving the JAK-STAT signaling pathway [12]. Activation of the IFN-receptor has been demonstrated to result in the differential induction of genes that protect against viral infection, regulate immune cell activity, control cell growth, and reduce oxidative stress [9, 12, 13]. IFNs are an accepted treatment for hepatitis B, hairy cell leukemia, and malignant melanoma. In addition, Betaseron (IFN**β**-1b; Bayer HealthCare), a type I interferon, provided the first approved treatment for MS [14].

Chemokines are potent immune modulators that act through a specific interaction with G-protein coupled receptors (GPCR). Chemokine binding to GPCRs results in important biologic effects [15, 16]. Like IFNs, chemokines and their receptors play a direct role in the regulation of immune responses and are proven drug targets. As much as 2% of the entire human genome encodes genes related to GPCR functions, and close to 50% of all current drugs are modulators of GPCR activity [17, 18]. One important aspect of chemokines and their receptors is that they play a central role in the regulation of immune cell migration to sites of inflammation. Chemokine-dependent activation of GPCRs causes the activation of heterotrimeric G-protein subunits, leading to regulation of enzymes and ion channels that results in enhanced cell migration and adhesion [18–21]. A recently discovered protein family of negative regulators of G-protein signaling (RGS) controls, in part, the extent of G-protein subunit activation. RGS proteins terminate G-protein signaling by accelerating GTP hydrolysis of activated G-protein subunits [20, 21]. Upregulation of certain RGS proteins has been shown to decrease immune cell migration and reduce chemokine-dependent calcium flux, an established measurement of GPCR signaling. RGS activity appears to be tightly controlled and highly dependent on tissue distribution and expression. 

Although the coordinated response of cytokines and chemokines is known to be important, studies focused on identifying mechanisms by which these two families of immune modulators can work together to elicit an appropriate immune response are limited. In this paper, we describe for the first time the induction of a negative regulator of G-protein signaling 1 (RGS1) by IFN-*β*, suggestive of a functional link between IFN and chemokine signaling.

## 2. Materials and Methods

### 2.1. RGS1 Expression in Human PBMCs and Immune Cell Subpopulations

Peripheral blood mononuclear cells (PBMCs) were obtained from healthy individuals and verified to be free of viral infection (All Cells LLC, Berkeley, CA). RNA was isolated using Qiagen RNeasy as previously described [13]. PBMCs (1 × 10^6^ cells/mL) were cultured (37°C, 5.0% carbon dioxide (CO_2_)) and grown in Eagle's Minimum Essential Medium supplemented with 10% v/v heat-inactivated fetal bovine serum and 2.0 mM L-glutamine. Antibiotics were not included in the cell culture media. Cells were incubated with or without IFN-*β*-1b (1000 International Units (IU)/mL, 1 × 10^7^ cells), IFN-*γ* (R&D Systems, Minneapolis, MN; 1000 IU/mL, 1 × 10^7^ cells) or phorbol myristate acetate (PMA) (Roche Diagnostics, Indianapolis, IN; 2.0 ng/mL, 1 × 10^7^ cells) for the times indicated. IFN-*β*-1b concentrations used for cell stimulation in culture media were below the pharmacological dose. Cells were collected, washed twice in cold phosphate-buffered saline (PBS), and RNA was isolated. To determine the specificity of induction of RGS1 by IFN-*β*, PBMCs were stimulated with or without IFN-*β* (1000 IU/mL, 1 × 10^7^ cells) for either 4 or 18 hours. RNA isolated at each time point was analyzed by reverse transcription polymerase chain reaction (RT-PCR; TaqMan) using specific RGS primer sets (RGS1, Hs00175260_m1; RGS2, Hs00263639_m1; RGS5, Hs00186212_m1; RGS7, Hs00243156_m1; RGS12, Hs01017940_m1; RGS14, Hs00197761_m1) (Applied Biosystems, Foster City, CA). 

Primary cells including B cells, monocytes, and T cells were isolated by negative selection using MACS cell separation (Miltenyi Biotec Inc., Germany) [22]. Purity of each cell population was determined to be greater that 90% by measurement of cell-type-specific surface markers using fluorescent-activated cell sorting (FACS) (data not shown). Isolated cell populations (1 × 10^6^ cells/mL) were incubated with or without IFN-*β* (1000 IU/mL) for 4 hours. After stimulation, cells were washed in cold PBS, RNA isolated, and RGS1 expression determined by TaqMan.

### 2.2. IFN Receptor-Dependent Induction of RGS1

The concentration of antisera needed to neutralize the IFN receptor was determined using a previously characterized IFN-dependent reporter assay [10]. Generation of a rabbit polyclonal antisera recognizing the ectodomain of the IFN receptor (IFNAR2c) has been previously described [11]. A stable cell line (HT1080LUC) containing an IFN reporter was preincubated with increasing concentrations of either preimmune or IFNAR2c-neutralizing antisera for 1 hour at room temperature. Cells were then stimulated, with or without IFN-*β*, for 4 hours, and reporter activity was measured after cell lysis using a luciferase substrate (Luciferase Assay System, Promega, Madison, WI). The amount of IFNAR2c antisera needed to inhibit activation of the IFN-receptor was determined relative to preimmune sera. 

PBMCs (1 × 10^6^ cell/mL) were incubated for 1 hour at room temparature in cell-culture media containing preimmune sera or IFNAR2c antisera at a concentration able to neutralize IFN-receptor activity by >95%. Cells were stimulated with or without IFN-*β* (1000 IU/mL) for 4 hours followed by cell lysis and RNA isolation as previously described. IFN-*β*-dependent expression of RGS1 in PBMCs treated with preimmune sera, IFNAR2c-neutralizing sera or control (unstimulated) samples was determined by TaqMan as previously described.

### 2.3. Patients

MS patients were diagnosed with definite MS according to the McDonald criteria and recruited for this study as previously described [13, 23]. Patients had an Expanded Disability Status Scale score of 2.5 ± 1.2; and all patients exhibited characteristic magnetic resonance imaging (MRI) lesions and two or more episodes of clinically evident relapses and remissions. Patients were continually monitored during the course of the study and were free of exacerbations for at least 3 months before and after blood collection. Study approval and written consent information were acquired and documented as described previously [13]. The patient cohort used in this study was held naïve to immunosuppressants or corticosteroids treatment for 1 year prior to, and during, the study. 

MS patients were under treatment with Betaseron (IFN-*β*) following an established clinical protocol for an average of 1.8 ± 0.8 years before beginning the study. Prior to the first sample draw, therapy was discontinued for 64 hours. After 64 hours a baseline sample (*t* = 0) was drawn, then a standard dose of IFN-*β* (8 million IU, specific activity 2.3 × 10^7^ IU/mg) was self-administered subcutaneously under physician supervision, and heparinized blood samples drawn at 4, 18, and 42 hours post-IFN-*β* administration. PBMC and RNA isolation was performed as previously described [13]. 

### 2.4. GeneChip and Computational Analysis

PBMCs were isolated using Ficoll-Paque Plus (Amersham Biosciences, NJ), and purified RNA characterized using an Agilent 2100 bioanalyzer (Agilent Instruments, Inc., Foster City, CA) [13]. RNA was profiled using Affymetrix oligonucleotide microarrays (Affymetrix GeneChip HG-U133A; Affymetrix Inc., Santa Clara, CA). Gene expression values were determined using Bioconductor's implementation of the Robust Multichip Average (RMA) algorithm with default options [13, 24, 25]. Gene expression resulting from Affymetrix probe-set measurements are presented as RMA computations performed with quantile normalization, including standardization and background adjustment [13, 25]. GeneChip probe sets are classified as 25-mer oligonucleotide sequences (*n* ~ 11) that hybridize to the corresponding nucleotide sequence present in a given gene. Gene expression analysis was also performed with the Statistical Analysis and Critical Rationalization of Expression Data (SACRED) software analysis package developed at Berlex Biosciences, Inc. (Richmond, CA).

### 2.5. Confirmation of RGS1 Expression in MS Patients

General methods for performing quantitative RT-PCR (qRT-PCR) have been previously described [13]. Briefly, expression of RGS1 was measured using RGS1-specific primer sets (Hs00175260_m1) (TaqMan Gene Expression Assays, Applied Biosystems, Foster City, CA). RNA samples were isolated using RNeasy Midi endotoxin-free Kit (Qiagen, Inc., Santa Clara, CA) and run in triplicate or quadruplicate for each sample. Analyzed RNA samples were identical to those used for gene chip analysis. PCR reactions were performed using a 96-well Opticon II DNA Engine (BioRad, Hercules, CA) or an ABI Prism 7900HT Sequence Detection System (Applied Biosystems, Foster City, CA,). Results are presented as linearized *C*(*t*) values normalized to glyceraldehyde-3-phosphate dehydrogenase (GAPDH).

### 2.6. Immunoprecipitation and Immunoblotting of RGS1

RGS1 polyclonal antisera used for immunoprecipation was generated against the 36 N-terminal amino acids of RGS1 (Harlan Laboratories, San Francisco, CA). The amino acid sequence MFFSANPKELKGTTHSLLDDKMQKRRRPKTFGMDMKA was coupled to KLH and used to produce RGS1 antisera in rabbits. The immunizing RGS1 peptide sequence was used as an input sequence to query numerous databases using the NCBI Basic Local Alignment Search Tool (BLAST) for detecting overlapping sequence alignments. Using this approach, the immunizing peptide sequence was shown to be specific for RGS1 or RGS1 isoforms with only a minor overlap with a predicted sequence for a G-protein 3-like molecule in Oryctolagus cuniculus (European rabbit). RGS1 antisera was first analyzed by ELISA to demonstrate immune reactivity against the immunizing peptide coupled to bovine serum albumin. Evaluation of RGS1 antiserum was further appraised by immunoblotting using purified RGS1 containing a 6xhistidine TAG at the C-terminus [26]. 6xHis RGS1 (5 *μ*g/200 *μ*L sample buffer) was subjected to SDS-PAGE using a large single well comb. Following SDS-PAGE, proteins were transferred to a polyvinylidene difluoride membrane (PVDF) (Pro-Blot, Appled Biosystems, Inc.) and incubated in blocking buffer (20 mM Tris-HCL (pH 7.3), 150 mM NaCl containing 0.1% Tween 20) overnight at room temperature. The resultant membrane was then cut into individual strips, and each strip was placed in its own well using an 8 well plastic tray. Membrane strips were probed with either preimmune sera, RGS1 antisera, or with a specific antibody recognizing 6xHis (6xHis Tag antibody-HRP, Ab1187) (Abcam,Cambridge, MA). Strips probed with the 6xHisTag antibody-HRP (1 : 1000 dilution) or RGS1 antisera (1 : 1000 dilution) or preimmune sera (1 : 1000 dilution) were incubated for two hours at room temperature and washed. After washing the membrane strips in blocking buffer, the strips incubated with RGS1 or preimmune antisera were further incubated with a second antibody (goat anti-rabbit IgG-HRP; 1 : 2000 dilution) (Abcam, Cambridge, MA) for 1 hour at room temperature followed by washing in blocking buffer. All membrane strips were arranged next to each other on a plastic sheet, and RGS1 (or 6xHisTagged RGS1) was detected using a chemiluminescent detection method (Supersignal West Pico Chemiluminescent Kit, Pierce/Thermo Scientific, Rockford, IL).

For immunoprecipation of RGS1, PBMCs were incubated with or without IFN-*β* (1000 IU/mL), IFN-*γ* (1000 IU/mL), or PMA (2 ng/mL) for 20 hours. An equal number of cells (2 × 10^8^) were collected by centrifugation (2000 × g, 10 minutes), washed twice in cold PBS, and lysed in solubilization buffer (20 mM Tris-HCl (pH 8.0) buffer containing 150 mM sodium chloride (NaCl), 1.0% Nonidet P-40 (v/v), and 2 mM EDTA) at 4°C. Insoluble material was removed by centrifugation (10 minutes, 10 000 × g, 4°C) and RGS1 antiserum (1 : 500) (described above) or preimmune (PI) (1 : 500) sera added to each lysate. Lysates were incubated overnight at 4°C, mixed with Protein-G agarose (Boehringer Ingelheim, Inc.), incubated for 1 hour, washed in blocking buffer, and bound material (immunoprecipate) removed by incubation with SDS-PAGE sample buffer. Protein-G agarose beads were removed by centrifugation and the remaining supernatant resolved by sodium dodecyl sulfate polyacrylamide gel electrophoresis (SDS-PAGE; 10%-20% Tris-Glycine, Invitrogen, Inc.) [11]. After electrophoresis, the resolved protein was transferred to a PVDF membrane and incubated overnight at room temperature in blocking buffer. 

The membrane was then incubated with a specific chicken RGS1 antisera purchased from ProSci Inc, CA (1 : 2000 dilution) for two hours at room temperature, washed in blocking buffer, and incubated with a horseradish peroxidase (HRP)-conjugated second antibody (anti-chicken IgY-HRP; 1 : 1000 dilution, ProSci Inc, CA). Immunoprecipated RGS1 was detected using a chemiluminescent detection method as previously described. Protein load was calibrated to cell number and protein concentration. After transfer to PVDF, transferred protein was also viewed using the reversible protein stain Ponceau S (Thermo Scientific, Rockford, IL).

### 2.7. Measurement of Chemokine Receptor Expression and Apoptosis Using FACS

A human monocytic cell line (THP-1) in active growth phase was incubated with or without IFN-*β* (1000 IU/mL) for 20 hours. Cells (1 × 10^7^/mL) were collected, washed in cold PBS, and incubated on ice for 1 hour with a formyl peptide receptor (FPR)-phycoerythrin or CXCR4-phycoerythrin-conjugated antibody (FPR-PE or CXCR4-PE) (Abcam, Inc. Cambridge, MA). Apoptosis was determined by measuring the extent of expression of the apoptosis marker annexin-PE relative to the viable cell marker 7-amino-actinomycin D (7-AAD). FACS was performed using a Beckman-Coulter Cytomic FC 500 MPL and CMP data analysis software (Beckman-Coulter, Fullerton, CA).

### 2.8. Chemotaxis Assay

Calcein AM-labeled (Molecular Probes, Carlsbad, CA) primary monocytes and THP-1 cells were assessed for their ability to migrate using 5-*μ*m pore size Multiscreen MIC filter plates (Millipore, Temecula, CA). One hundred fifty microliters of migration buffer (HBSS plus Ca^2+^/Mg^2+^, 0.1% BSA) or buffer plus SDF1*α*, MCP1, or MCP2 (R&D Systems, CA) was placed in the bottom chamber. One hundred thousand IFN-*β* or mock-treated cells in migration buffer (100 *μ*L) were gently overlaid on the top chamber. The culture plates were incubated at 37°C, 5% CO_2_ for 2–4 hours, and cells migrating to the bottom chamber were quantified on a Flexstation II calibrated to measure endpoint fluorescence (488 nm excitation and 538 nm emission). Results are shown as percentage of migration relative to the original number of cells applied. 

### 2.9. Statistics

The mean values ± standard deviations (SD) were determined for each given experiment. Statistical tests comparing differences between groups were performed using the Student's *t*-test. A *P*-value of <.05 was considered to be significant.

## 3. Results

### 3.1. Specificity of RGS1 Induction by IFN-*β*

**Figure 1:**
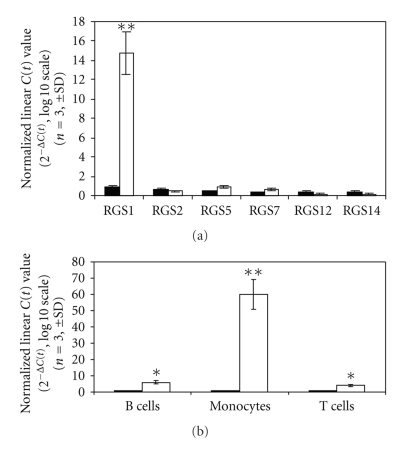
RGS1 expression and cell-type specificity. PBMCs were stimulated with IFN-*β* (1000 IU/mL) for 4 hours (Figure 1(a)). RNA was isolated, and expression of different RGS family members was determined by RT-PCR as described in Section 2. Results are presented as linearized *C*(*t*) values (*n* = 3, ±SD) normalized to GAPDH. For comparison, samples having background expression were calibrated to 1.0 (black columns). RGS1 was clearly the most predominant RGS family member induced by IFN-*β* after 4 hours (white columns). Each RGS family member tested is shown on the *x*-axis. Figure 1(b) shows the IFN-*β*-dependent expression of RGS1 in three different primary cell populations (B cells, monocytes, T cells). Data are presented as nonlinearized *C*(*t*) values normalized to GAPDH (mean ± SD, *n* = 3). *P < .05,**P < .01.

RGS1 is a member of a large family of regulators of G-protein signaling, the R4 RGS proteins [27]. RGS proteins are expressed in a number of different cell types. RGS1 is known to be expressed in immune cells, and overexpression in B-cells attenuates chemokine signaling [28]. In this study, the specific induction of RGS1 by IFN-*β* was first observed in PBMCs (Figure 1). IFN-*β* was shown to preferentially induce the expression of RGS1 over that of other RGS family members including RGS2, RGS5, RGS7, RGS12, and RGS14 (Figure 1(a)). IFN-*β*-dependent induction of RGS1 was next determined in three purified immune cell populations isolated to homogeneity (greater than 90%) using negative selection [22]. Induction of RGS1 by IFN-*β* was shown to occur in B cells, monocytes, and T cells (Figure 1(b)). Although all three cell types were responsive to IFN-*β* stimulation, RGS1 induction appeared to be strongest in monocytes. 

**Figure 2:**
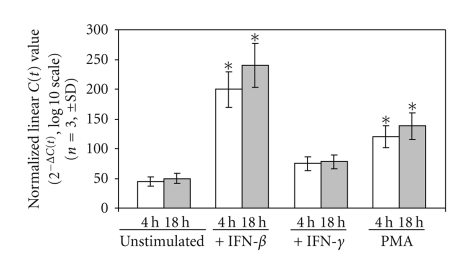
Induction of RGS1 by IFN-*β*, IFN-*γ*, and PMA. PBMCs obtained from healthy individuals were stimulated and RGS1 expression determined as described in Section 2. PBMCs were stimulated with IFN-*β* (1000 IU/mL), IFN-*γ* (1000 IU/mL), and PMA (2 ng/mL) for the times indicated (white column = 4 hours; gray column = 18 hours post-IFN-*β* stimulation). All results were normalized to GAPDH and expressed as the mean ± SD, *n* = 3. *P < .05.

Specificity and kinetics of induction of RGS1 were then evaluated. PBMCs were stimulated for 4 or 18 hours with either IFN-**β**, IFN-*γ*, or PMA followed by determination of RGS1 RNA expression (Figure 2). RGS1 expression was compared to the steady-state level observed in unstimulated cells. Results show a preferential induction of RGS1 by IFN-*β* relative to both PMA and IFN-*γ*. PMA induction of RGS1 expression was also previously described in the human monocytic cell line U937 [29]. 

**Figure 3:**
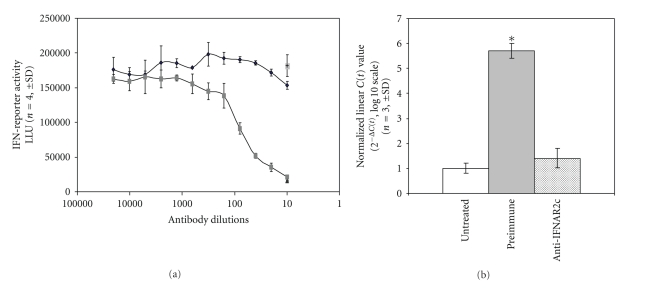
IFN-*β* induction of RGS1 is dependent on IFN-receptor activation. Neutralizing antisera recognizing the IFNAR2c IFN-receptor was incubated with PBMCs at various concentrations to determine the dilution necessary to achieve greater than 95% inhibition of an IFN-reporter assay (see Section 2). Figure 3(a): Cells (HT1080LUC) were incubated as previously described with preimmune (dark squares) or IFNAR2c antisera (gray squares). Antibody dilution is represented on the *x*-axis. The extent of IFN-reporter activation is shown on the *y*-axis represented as luciferase light units (LLU). IFN-reporter activity for IFN-*β*-stimulated HT1080LUC cells in the absence of antisera is also shown for comparison (single gray square). Data are presented as mean (*n* = 4) ±SD. Figure 3(b): TaqMan analysis of IFN-*β* induced expression of RGS1 in the absence (untreated and preimmune) or presence (anti-IFNAR2c) of an IFN-receptor neutralizing antisera. RGS1 induction is expressed as linearized *C*(*t*) values normalized to GAPDH (*n* = 3, mean ± SD). *P < .05.

To confirm a direct role for IFN-*β* in the induction of RGS1, RGS1 expression was determined in the presence of neutralizing antibody that blocks activation of the IFN receptor (Figure 3) [10]. The antibody concentration needed to neutralize the IFN receptor was determined using a cell line stably expressing an IFN-responsive reporter (HT1080LUC) (Figure 3(a)) [10]. HT1080LUC cells incubated with neutralizing antibody showed a strong inhibition of RGS1 induction after IFN-*β* stimulation relative to preimmune sera and untreated cells (Figure 3(b)). These results demonstrate that the specific induction of RGS1 by IFN-*β* is dependent upon activation of the IFN receptor.

### 3.2. Expression of RGS1 in MS Patients

**Figure 4:**
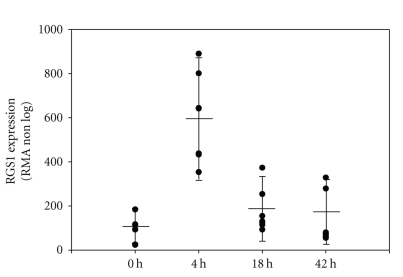
Induction of RGS1 in MS patients after a single dose of Betaseron. Seven patients with clinically defined MS were selected for study. Patients had been on treatment for an average of 1.8 ± 0.8 years and were free of exacerbations for 3 months before and after blood collection. PBMCs were isolated and RNA expression analyzed using GeneChip technologies described in Section 2. Betaseron induction of RGS1 gene expression is presented as RMA values. RGS1 (Affymetrix RGS1 probe set 202988_s_at) was demonstrated to be strongly up-regulated reaching a maximum level 4 hours after administration of Betaseron. The time of blood draw (0 hours = wash-out period before administration of Betaseron, and 4, 18, and 42 hours post-Betaseron administration) is shown on the *x*-axis. RGS1 expression (RMA non-log) is presented as a scatter plot showing the response of each patient including the mean expression (solid bar) for all MS patients at each time point. All patients received a pharmacological dose of Betaseron (250 *μ*g/mL; 8 M IU).

**Figure 5:**
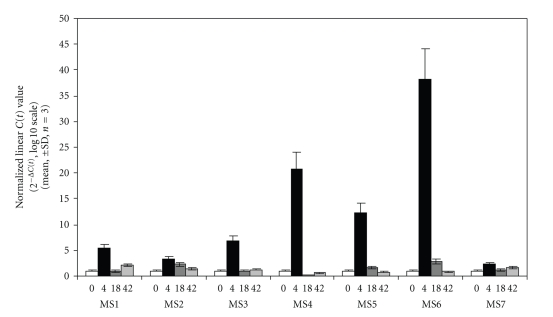
Confirmation of RGS1 GeneChip expression data using RT-PCR. The same RNA obtained from MS patients treated with Betaseron and used for the GeneChip study was used to independently validate the expression of RGS1 using RT-PCR. RGS1 expression was determined using RT-PCR (TaqMan custom primer set Hs00175260_m1) at 0, 4, 18, and 42 hours post-Betaseron administration. MS 1–7 refers to MS patients included in the initial study. Data are expressed as linearized *C*(*t*) values normalized to GAPDH. Data are presented as the mean ± SD (*n* = 3).

Genetic risk factors for susceptibility to multiple sclerosis have recently been identified [30, 31], the most prominent being HLA DRB1. Interestingly, RGS1 was also included in one of the MS susceptibility loci identified. In this regard, it was important to determine if treatment of MS patients with IFN-*β* leads to changes in RGS1 expression. Based on previous pharmacokinetic studies, MS patients were asked to stop treatment for 64 hours to allow for a “washout” period in which IFN-*β* dependent gene expression would return to baseline levels. Patient blood was drawn just prior to administration of IFN-*β* and at 4, 18, and 43 hours post-administration. PBMCs were isolated, RNA purified and differential gene expression analyzed. As previously demonstrated, induction of a number of differentially expressed genes was observed in MS patients following administration of a single dose of IFN-*β* [13]. Examination of the differential gene expression pattern using RMA and SACRED demonstrated a transient change in differential gene expression that returned to near preadministration levels by 42 hours [13]. Upon further examination, we observed the upregulation of RNA encoding RGS1 in all seven MS patients studied (Figure 4). This result supported our initial in vitro observations and demonstrated the regulation of RGS1 by IFN-*β* in MS patients. In MS patients, the expression of RGS1 reached a maximum level 4 hours post-administration of IFN-**β** and returned to near baseline by 42 hours. A similar time course of induction of RGS1 occurred in each patient; however, patient-to-patient variability in the magnitude of response was observed (Figure 4). Variability of response was not due to differences in RNA quality or method of GeneChip analysis [13]. To validate the observed expression of RGS1 in MS patients, we performed an RGS1-specific TaqMan assay making use of the same RNA samples used for the initial Affymetric GeneChip analysis. Using this approach, we also observed patient-to-patient variability in magnitude of response for the same samples, and a maximum expression of RGS1 at 4 hours post-administration of IFN-*β* followed by a return to near baseline by 42 hours (Figure 5).

### 3.3. IFN-*β* Treatment Increases RGS1 Protein Expression

**Figure 6:**
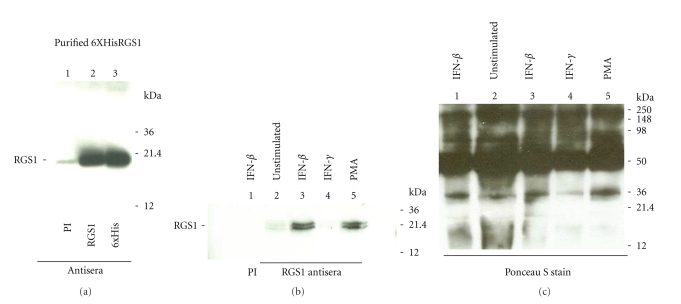
Immunoprecipation of RGS1 from human PBMCs. Purified 6xHisRGS1 was used to evaluate RGS1 immune sera (a). 6xHisRGS1 was subjected to SDS-PAGE followed by immunoblotting as described in Section 2. The resultant immunoblot was probed with either preimmune sera (PI), RGS1 immune sera, or a specific antibody recognizing 6xHis. Lane 1, preimmune sera (PI); lane 2 RGS1 antisera (RGS1); lane 3 6xHis antisera (6xHis). (b) PBMCs were stimulated for 20 hours with either IFN-*β* (1000 IU/mL); IFN-*γ* (1000 IU/mL), or PMA (2 ng/mL). RGS1 was immunoprecipated using cell lysates obtained from equal amounts of cells (2 × 10^8^ cells/sample) and a normalized protein concentration. Immunoprecipated RGS1 was detected by immunoblotting using RGS1-specific antisera as described in Section 2. Lane 1, preimmune sera IFN-*β*-stimulated; lane 2, RGS1 antisera (unstimulated cells); lane 3, RGS1 antisera (IFN-*β*-stimulated cells); lane 4, RGS1 antisera (IFN-*γ*-stimulated cells); lane 5, RGS1 antisera (PMA-stimulated cells). The location of RGS1 (23 kDa) is indicated (RGS1). (c) Ponceau S staining of the PVDF membrane to visualize efficiency of protein transfer and load. Molecular weight markers are also included (kDa).

RNA expression represents the first step in the production of a functional protein. RNA induction generally precedes that of protein expression although changes in RNA half-life or stability could possibly alter the level of RNA observed in the cell without resulting in an increased level of protein expression. Therefore, demonstrating that the induction of RGS1 RNA resulted in an increase in protein expression was important. The ability of RGS1 antisera to recognize RGS1 was initially determined by immunoblotting using purified 6xHisRGS1 (Figure 6). 6xHisRGS1 was recognized by either RGS1 antisera (Figure 6(a), lane 2) or an antibody directed against 6Xhistidine (Figure 6(a), lane 3). PBMCs obtained from healthy individuals were stimulated with IFN-*β*, IFN-*γ*, or PMA for 20 hours. For normalization of protein levels, cell numbers were made identical and an equal protein concentration was used for each immunoprecipitation. Cells were lysed and RGS1 was immunoprecipitated using an RGS1 polyclonal antisera. As shown in Figure 6(b), RGS1 was strongly induced by IFN-*β* stimulation and clearly present in immunoprecipitates from IFN-*β-* and PMA-stimulated cells (Figure 6(b), lanes 3 and 5). RGS1 was not detected using preimmune sera (Figure 6(b), lane 1) and RGS1 was not appreciably detected in RGS1 immunoprecipitates using cell lysates made from PBMCs stimulated with IFN-*γ* (Figure 6(b), lane 4). However, a faint protein doublet was observed in both unstimulated and IFN-*γ* stimulated cells (Figure 6(b), lanes 2 and 4). This protein doublet likely represents the basal level of RGS1 expression in this cell population. The appearance of RGS1 as a protein doublet of approximately 23 kDa is consistent with its expected molecular weight and previous observations [29]. It is unclear why immunoprecipitated RGS1 appears as a doublet. This may occur due to an amino acid truncation, misfolding of the protein, or the presence of protein isoforms.

### 3.4. Inhibition of Cell Migration by IFN-*β*

**Figure 7:**
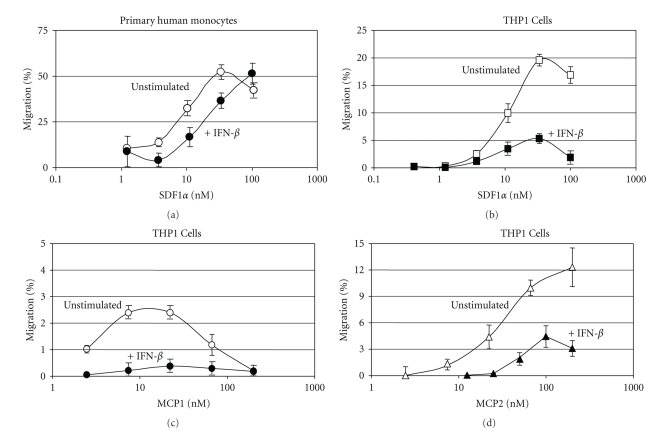
Effect of IFN-*β* on chemokine-dependent migration of monocytes. Monocyte transmigration across an artificial barrier in response to chemokine stimulation was determined as described in Section 2. Primary monocytes incubated with IFN-*β* (1000 IU/10^7^ cells) for 20 h were stimulated with various concentrations of SDF1*α* (Figure 7(a)). IFN-*β*-stimulated (dark circles) and unstimulated (open circles) cells are shown. The concentration of SDF1*α* (nM SDF1*α*) is shown on the *x*-axis along with transmigration, as percent (% migration) of cells applied, on the *y*-axis. The monocytic cell line THP-1 was incubated with IFN-*β* (1000 IU/10^7^ cells) for 20 hours and then stimulated with increasing concentrations of either SDF1*α* (Figure 7(b)), MCP1 (Figure 7(c)), or MCP2 (Figure 7(d)). The concentrations of SDF1*α*, MCP1, and MCP2 are shown on the *x*-axis (nM chemokine) and results are presented as percent of total cell migration (% migration). Data represents mean ± SD of *n* = 4.

Previous studies have documented the role of IFN-*β* in immune modulation and expression of Th1 and Th2 cytokines [9, 12]. Less well documented is the role of IFN-*β* in the regulation of immune cell migration, a process in which chemokines, GPCRs, and RGS proteins play a central role. While a limited number of studies have demonstrated that IFNs induce shedding of adhesion molecules from the cell surface [32, 33], few studies describe a role for IFNs directly in immune cell migration. In this regard, the upregulation of RGS1 by IFN-*β* would be expected to result in a reduction in chemokine-dependent cell migration. To determine if such an association exists, we stimulated monocytes with IFN-*β* for a time period demonstrated to sufficiently induce RGS1 (Figure 6). A chemokine-dependent transmigration assay was then used to measure the degree of cell migration. Both primary monocytes (Figure 7(a)) and the monocytic cell line THP-1 (Figures 7(b), 7(c), and 7(d)) were shown to migrate across an artificial barrier when stimulated with either SDF1*α*, MCP1, or MCP2. In all cases, stimulation of monocytes or THP-1 cells with IFN-*β* for the time required to elevate levels of RGS1 inhibited monocyte transmigration relative to untreated cells. Inhibition of chemokine-dependent cell migration by IFN-*β* was dose dependent and consistent with the time and concentration required for inhibition of SDF*1α*-dependent calcium flux (data not shown). These results provide a direct measurement of the effect of IFN-*β* on cell migration but only indirectly link the upregulation of RGS1 to regulation of cell migration.

### 3.5. Effects of IFN-*β* on Chemokine Receptor Expression and Cell Growth

**Figure 8:**
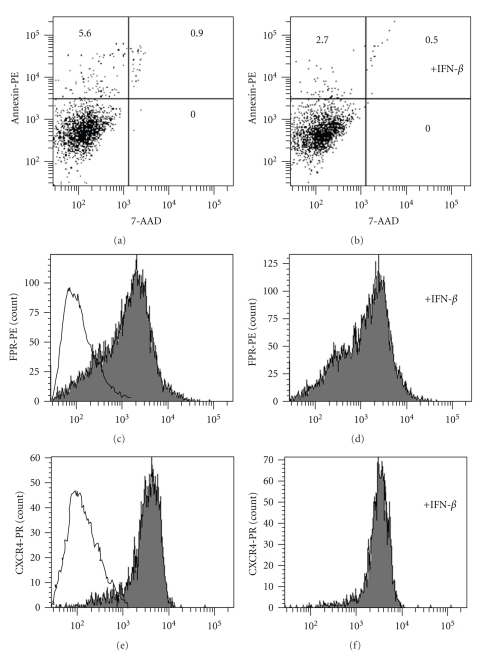
IFN-*β* inhibition of calcium flux is not due to apoptosis or chemokine receptor down regulation. Monocytes were incubated with IFN-**β** for 20 hours, and the degree of apoptosis in untreated and IFN-*β* treated monocytes was determined by measuring Annexin-PE staining relative to the cell viability dye 7-AAD as described in Section 2 (a), unstimulated; (b), IFN-*β*-stimulated. Annexin-PE fluorescence is shown on the *y*-axis and 7-AAD on the *x*-axis. The percent (%) fluorescent events (total of 10 000 measurements) are shown in each quadrant. CXCR4 and FPR expression was determined using FACS as described in Section 2. FPR expression levels are shown for control (solid line) and unstimulated (c) and IFN-*β*-stimulated (d) cells. CXCR4 expression levels are also shown for unstimulated (e) and stimulated cells (f). Cells were incubated for 20 hours with IFN-**β** prior to measuring CXCR4 and FPR expression levels.

It is known that IFNs can affect cell surface receptor expression and induce apoptosis. Such effects could presumably give rise to indirect changes in GPCR levels and thereby influence RGS expression. Therefore, it was important to show that IFN-*β* used as described in these studies, did not influence GPCR expression or induce apoptosis. When using the apoptosis marker Annexin, no difference in apoptosis was observed between untreated monocytes (Figure 8(a)) and those treated with IFN-*β* (Figure 8(b)). In terms of GPCR expression, cell-surface expression of two GPCRs (CXCR4 and FPR) representing different GPCR families analyzed. Formyl peptide receptor (FPR) [34] expression showed no difference in cell-surface expression in the absence (Figure 8(c)) or presence (Figure 8(d)) of IFN-*β* treatment. A similar lack of affect of IFN-*β* on the cell-surface expression of CXCR4 [35] was also observed (Figures 8(e) and 8(f)).

## 4. Discussion

Chemokines play a major role in the trafficking of immune cells to sites of inflammation, in and out of germinal centers located in lymphoid tissues, and in the repair of damaged tissue. Migration of immune cells is mediated by a complex interplay between cell-surface adhesion molecules and chemoattractant receptors and ligands [34–36]. Cytokines, a family of immune modulators different from chemokines, play a central role in mediating both innate and adaptive immunity and protection from viral infection [12, 34, 35]. Harmonizing the interplay between chemokines and cytokines appears essential for the establishment of an effective and properly regulated immune response. 

RGS proteins regulate chemokine-induced activation of GPCRs by accelerating the GTPase activity associated with the G-protein alpha subunit. The RGS protein family consists of 30 or more distinct proteins containing one or more RGS domains. In mammalian cells, RGS proteins interact with other molecules involved in signal transduction including receptors, phospholipids, and scaffolding proteins [19–21, 27]. RGS proteins are highly regulated at the transcriptional level and have unique tissue distribution. Some RGS members like RGS2 and RGS3 have a wide distribution of expression while others like RGS4, RGS6, RGS7, RGS8, RGS9, RGS10, RGS11, and RGS14 are only found in a specific organ or tissue. RGS1 appears to be highly expressed in lymphocytes, suggesting a role for RGS1 in immune cell regulation. In support of this role, previous studies using RGS knockout mice suggest a strong link between RGS1 expression and B cell migration [28]. 

Results described in this study identify a possible additional role for IFN-**β** in the regulation of immune cell function. Our findings demonstrate that IFN-*β* may influence chemokine-dependent signaling though induction of a negative regulator of G-protein signaling. Regulating immune cell trafficking to sites of inflammation or, as in MS, inflammatory lesions in the central nervous system (CNS), goes hand in hand with immunological properties previously associated with type I IFNs [9, 12, 32, 37–41]. What is surprising is the observation that IFN-*β* may influence regulation of GTPase activity. Previous studies have described a role for IFN-*β* in regulating immune cell migration by enhancing shedding of adhesion molecules from the cell surface [33]. IFN-*β* regulation of RGS1 points to an additional means by which IFNs can exert an inhibitory effect on chemokine-dependent lymphocyte migration. Such a mechanism can help to explain the previously described involvement of IFNs in the regulation of immune cell migration across human brain microvascular endothelial cell monolayers [42], the transverse of T cells through basement membranes [43], and human lymphocyte transmigration [44]. 

Our observations identify IFN-*β* as a regulator of RGS1 expression in lymphocytes. It should be noted that IFN-*β* regulation of RGS1 alone may not be sufficient to regulate the full extent of ligand-dependent GPCR activation of G-proteins. It is likely that other proteins interact with RGS1 or affect the regulation of additional RGS proteins to fine tune specific activation and migration of immune cells during inflammation. Recent studies have demonstrated that upregulation of RGS1 reduces the migration of regulatory T-cells (Tregs) relative to naïve T cells [45]. In this regard, G-protein regulation by RGS1 and other RGS family members can be highly cell-specific and involves other unidentified factors [46, 47]. Such specificity would also suggest that RGS control of immune cell responsiveness would be dependent on the presence of a given GPCR.

Translating in vitro observations made in cell culture to in vivo responses observed directly in a disease setting can provide valuable insights into the possible mechanism of action of a drug. In addition, the identification of an RGS1 genetic risk factor susceptibility locus in MS suggests a possible role for single-nucleotide-polymorphism-derived RGS1 variants in disease susceptibility. Therefore, it is interesting to note that RGS1 is upregulated in IFN-*β* treated MS patients perhaps in an effort to compensate for an abnormal RGS1. A hallmark of MS is the development of inflammatory lesions in the CNS. Formation of inflammatory lesions in MS patients as recognized by MRI using gadolinium enhancement is a measurement of CNS inflammation associated with MS [48]. Formation of lesions within the CNS is thought to be mediated, in part, by the migration of activated immune cells from the periphery into the CNS. Once in the CNS, they interact with local antigen-presenting cells including astrocytes and microglia to further drive formation of inflammatory lesions visible by MRI. In addition, suppression of immune responses in MS patients has been linked to a reduction in the levels of oxidative stress, which, when left unchecked, can lead to neurodegeneration and axonal loss commonly observed in MS [49, 50]. The observed in vitro upregulation of RGS1 by IFN-*β* provided the opportunity to study the in vivo regulation of RGS1 directly in MS patients treated with IFN-*β*. Although only seven individual patents were studied, all patients showed significant upregulation of RGS1 expression after IFN-*β* administration. Studies are underway to confirm this observation using a larger cohort of MS patients. The upregulation of RGS1 as measured by gene expression profiling in MS patients highlights the possible importance of RGS1 for the regulation of chemokine activity in the treatment of MS. A single study such as this is not sufficient to demonstrate RGS1 regulation by IFN-*β* provides a new sole mechanism of action, nor is the effect likely unique to MS. However, such a mechanism could help to explain the observed inhibitory effects of IFN-*β* on migration of activated immune cells from the periphery to sites of inflammation within the CNS. 

IFN-*β* inhibition of cell migration demonstrates a role for IFN-*β* in the regulation of immune cell migration. RGS1 upregulated by IFN-*β* would be expected to enhance immune cell migration by accelerating GTPase activity, negatively regulating G-protein coupled receptor signaling. IFN-*β* was demonstrated to inhibit monocyte migration across an artificial barrier in response to chemokine. Inhibition of cell migration coincided with the time required to upregulate RGS1 in immune cells, suggesting an association between inhibition of cell migration and expression. Unfortunately, we were unsuccessful in identifying an siRNA able to downregulate RGS1 expression in these cells. Therefore, a direct link between IFN-*β* induced RGS1 and cell migration will require further studies. A RGS1 murine knockout is available and could provide an additional means to studying the role of IFN-*β* in regulation of RGS1 and cell migration. IFN-*β* did not appear to affect GPCR receptor expression or induce apoptosis in immune cells using conditions described in this study. Therefore, the observed reduction in monocyte cell migration was not due to an induction of cell death or a loss of G-protein coupled receptors from the cell surface.

The observation that a cytokine such as IFN-*β* can influence chemokine-dependent G-protein activation suggests a possible mechanism for crosstalk between these two important families of immune regulators. Studies are underway to further understand the relationship between IFN-*β*, GPCRs and RGS proteins with regard to control of immune cell surveillance, maturation, migration, and inflammation.
